# Synthesis of a precursor of D-fagomine by immobilized fructose-6-phosphate aldolase

**DOI:** 10.1371/journal.pone.0250513

**Published:** 2021-04-22

**Authors:** Gerard Masdeu, Luis Miguel Vázquez, Josep López-Santín, Gloria Caminal, Slavko Kralj, Darko Makovec, Gregorio Álvaro, Marina Guillén

**Affiliations:** 1 Department of Chemical, Biological and Environmental Engineering, Universitat Autònoma de Barcelona, Bellaterra, Barcelona, Spain; 2 Institute of Advanced Chemistry of Catalonia, IAQC-CSIC, Barcelona, Spain; 3 Department for Materials Synthesis, Jožef Stefan Institute, Ljubljana, Slovenia; Queen’s University Belfast, UNITED KINGDOM

## Abstract

Fructose-6-phosphate aldolase (FSA) is an important enzyme for the C-C bond-forming reactions in organic synthesis. The present work is focused on the synthesis of a precursor of D-fagomine catalyzed by a mutant FSA. The biocatalyst has been immobilized onto several supports: magnetic nanoparticle clusters (mNC), cobalt-chelated agarose (Co-IDA), amino-functionalized agarose (MANA-agarose) and glyoxal-agarose, obtaining a 29.0%, 93.8%, 89.7% and 53.9% of retained activity, respectively. Glyoxal-agarose FSA derivative stood up as the best option for the synthesis of the precursor of D-fagomine due to the high reaction rate, conversion, yield and operational stability achieved. FSA immobilized in glyoxal-agarose could be reused up to 6 reaction cycles reaching a 4-fold improvement in biocatalyst yield compared to the non-immobilized enzyme.

## Introduction

Iminocyclitols are iminosugars that act as inhibitors of intestinal glycosidase. This biological activity is of high pharmaceutical interest since these compounds can be used to modulate the postprandial glucose concentration, reducing the risk of developing insulin resistance [1]. D-fagomine, which presents the molecular configuration of D-glucose and D-mannose on carbons 3, 4 and 5, is the only iminocyclitol that can be obtained from a traditional food source, the seeds of buckwheat *(Fagopyrum esculentum)*. It is also present in other plant sources such as mulberry (Morus Alba, Moraceae) leaves and gogi (*Lycium chinense*) roots [2]. This compound, as other iminocyclitols, is able not only to quench and delay the hyperglycemic response to oral sucrose and starch but to selectively agglutinate pathogenic enterobacteria in the intestine and to facilitate the adhesion of probiotics [3].

A two-step chemo-enzymatic synthesis of D-fagomine has been described based on an aldol reaction catalyzed by D-Fructose-6-phosphate aldolase (FSA) from *Escherichia coli* [4–6]. FSA is a decameric protein of 230 kDa that accepts unphosphorylated dihydroxyacetone (DHA) as a donor substrate [7,8]. This is an advantage towards phosphorylated-DHA-dependent aldolases since i) DHA is cheaper than the phosphorylated compound and ii) the removal of the phosphate group from the final product is circumvented. FSA can tolerate different donor compounds like hydroxyacetone, 1-hydroxy-2-butanone and glycolaldehyde [9–12]. However, aiming to widen the applicability of this biocatalyst in industrial target reactions, several authors have worked on increasing the FSA promiscuity by genetic engineering [9–11,13–19]. Mutant FSA A129S, obtained by site-directed mutagenesis gave a strikingly improved kcat/K_M_ towards DHA, D-G3P and D-F6P and potential complementary synthetic abilities to those of FSA wild type [20]. Therefore, FSA A129S is considered a promising biocatalyst for industrial application in aldol reactions.

It is known that enzyme implementation at large scale requires high stability under industrial reaction conditions. Several strategies can be used to improve biocatalyst stability such as enzyme immobilization. This strategy can also facilitate the recovery and the reuse of the biocatalyst in multiple reaction cycles also improving the downstream processing [21,22]. The immobilization process requires preliminary studies of the enzymes and the supports with the aim of selecting the most suitable method. Mesoporous supports are typically used for enzyme immobilization due to i) the wide range of available matrixes, functional groups, sizes and porous diameters, ii) their generally easy preparation, and iii) their easy operational use. Different types of materials, considering both organic (e.g. agar, chitosan or alginate) and inorganic compounds (e.g. Eupergit) have been applied as carriers for enzyme immobilization [6,23–26]. Several properties are considered desirable for the carrier such as high specific surface, high chemical and mechanical robustness, high enzyme binding capacity and high compatibility with the reaction medium. However, there is no material fulfilling all these characteristics for all the biocatalytic processes. Thus, the most suitable carrier for the target process has to be found by testing different options [6]. In addition to the mesoporous supports, nanocarriers could be also considered since they have several advantages. The enzymes immobilized on nanoparticles follow Brownian movement when are dispersed in aqueous media, showing similar properties to the soluble enzyme. Nanoparticles demonstrate great properties for enzyme immobilization like high surface area, minimization of diffusional problems and good mechanical stability. However, nanomaterials usually suffer from high cost of fabrication and their recovery from the reaction medium tends to be difficult [27]. Magnetic nanoparticles are a specific type of nanomaterials designed for efficient recovery of the biocatalyst from the reaction mixture by means of straightforward magnetic separation. Due to the great properties of this kind of support, several enzymes have been immobilized on magnetic nanoparticles obtaining different biocatalysts successfully applied in synthetic reactions [28–32]. Superparamagnetic nanoparticles have been usually used to avoid their agglomeration due to magnetic dipole-dipole interactions. Nevertheless, at a small size (below ~20 nm) the magnetic force acting on the individual superparamagnetic nanoparticle in a magnetic field gradient is usually too weak to enable a quick separation of the biocatalyst from the reaction media. Aiming for an efficient separation, many superparamagnetic nanoparticles can be therefore assembled into larger magnetic nanoparticle clusters (mNC) [33,34].

Regarding FSA immobilization, some authors have reported successful methodologies based on an enzyme embedding in layered double hydroxide (LDH) nanoplatelets or by means of a modification of this method based on an encapsulation of FSA-LDH in a carrageenan polymer [35–37].

**Fig 1:**
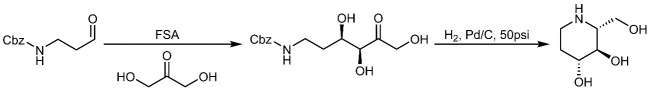
Enzymatic system for the synthesis of D-fagomine.

In the present work, the synthesis of (3*S*,4*R*)-6-[(Benzyloxycarbonyl)amino]-5,6-dideoxy hex-2-ulose, a precursor of D-fagomine (pre-D-fagomine), by soluble and immobilized FSA A129S has been studied (Fig 1). Several methods have been screened for FSA immobilization using both mesoporous carriers and magnetic nanoparticle clusters. The most promising immobilized derivatives have been tested for the synthesis of pre-D-fagomine, and their operational stability in subsequent reaction cycles has been evaluated.

## Materials and methods

### Materials

Recombinant FSA A129S was produced and purified according to the literature with an activity of 15.0 U mg^-1^ FSA (S1 Fig) [38]. *N*-(3-dimethylaminopropyl)-*N*’-ethylcarbodiimide (EDAC), α-glycerophosphate dehydrogenase–triosephosphate isomerase (GPD-TPI), D-fructose-6-phosphate (F6P) and 3-[(benzyloxycarbonyl)amino]propionaldehyde (β-CHO) were purchased from Sigma Aldrich (St. Louis, USA). Nicotinamide adenine dinucleotide (NADH) was purchased from BONTAC Bio-engineering (Shenzhen, China). 6% cross-linked agarose gels, MANA-agarose gels and High-density metal free sepharose were purchased from ABT-beads (Torrejón de Ardóz, Spain). Dihydroxyacetone (DHA) was purchased by Merck (Darmstadt, Germany). Iron (III) sulfate hydrate, iron (II) sulfate heptahydrate (ACS, 99%), citric acid (99%), tetraethoxysilane (TEOS, 99.9%), NH_4_OH (28–30%) were supplied by Alfa Aesar (Lancashire, UK). (Hydroxy(polyethyleneoxy)propyl) triethoxysilane (silane-PEG), triethoxysilylpropylmaleamic acid (silane-COOH), 3-mercaptopropyltrimethoxy silane (silane-SH), *N*-(trimethoxysilylpropyl) ethylenediaminetriacetate (silane-EDTA) were purchased by Gelest (USA). All other reagents were commercial products of analytical grade. Silane-C≡CH and silane-N_3_ were kindly supplied by Dr. Stane Pajk (Jožef Stefan Institute, Ljubljana, Slovenia) [39].

### FSA activity assay

The activity assay followed the cleavage of F6P to D-glyceraldehyde-3-phosphate (G3P) and DHA by using the auxiliary enzymes α-glycerophosphate dehydrogenase and triosephosphate isomerase (GPD-TPI) [8]. TPI converts G3P into DHAP, which is further reduced by GPD using NADH. FSA activity was monitored by the decrease in absorbance due to the conversion of NADH to NAD^+^ at 340 nm and 30 ^o^C with a UV/visible Cary 50 (Varian, Palo Alto, USA) spectrophotometer (Ɛ_340nm_ = 6.2 mM^-1^ cm^-1^). The reaction mixture (1 mL of total volume) contained 50 mM imidazole, 0.1 mM NADH, 5 mM F6P and 10 U mL^-1^ of GPD-TPI and 50 μL of the sample in distilled water under pH 8. One activity unit of FSA is defined as the amount of enzyme required for the conversion of 1 μmol of F6P per minute at pH 8.0, 30°C. The standard deviation of the FSA activity test was calculated from measurements performed by duplicate. Activity assays were carried out using 1.5 mL cuvettes suitable for UV. When the activity of immobilized FSA derivatives was measured, double of each volume was added and 3 mL cuvettes were used with magnetic stirring to maintain a proper suspension of the derivatives during the measurement.

For the determination of the low enzyme load at the mNC, an alternative activity test was proposed in this study. The activity was determined by following the DHA aldol addition reaction by HPLC. The immobilized enzyme was concentrated up to 50 μL by removing of supernatant using a magnetic field. This volume was added to the reaction mixture, containing 40 mM β-CHO and 100 mM DHA in 50 mM HEPES (pH 8.0), in a final volume of 1 mL. It was left for 90 min at 30°C, 1000 rpm of orbital stirring. The reaction was stopped by acidification (pH 2). After neutralization, the percentage of activity was determined by HPLC analysis of the preFagomine concentration. This test showed great linearity (S3 Fig).

### Enzyme immobilization onto functionalized mNC

Magnetic nanoparticle clusters (mNC) were obtained by the self-assembly of primary maghemite nanoparticles followed by coating of the magnetic clusters with a layer of silica (mNC-Si) (120 ± 15 nm mNC size, density 4300 kg m^-3^, 2 silane nm^2^) [40–42]. To enable the conjugation of the enzyme, mNC-Si was functionalized by grafting different functional silanes, such as silane-PEG, -NH_2,_ -COOH, -SH, -N_3_, -C≡CH, and -EDTA, onto their surfaces yielding mNC-PEG, mNC-NH_2,_ mNC-COOH, mNC-SH, mNC-N_3_, mNC-C≡CH, and mNC-EDTA, respectively. Functionalization procedures were described in detail elsewhere and the same strategy was followed for all silane bindings to silica surfaces [39,43]. Functionalized mNC are fully characterized and also commercially available by Nanos SCI company under trademark iNANOvative™.

For the preparation of mNC-CHO, 3 mg mNC-PEG was suspended for 19 h in 5 mL of a solution containing 64 mM NaOH, 28 mM NaBH_4_, 408 mM glycidol. NaIO_4_ (2 mM for experiments at pH 5.0; 90 mM for pH 8.0) was added to oxidize the aldehyde groups. Finally, mNC-CHO was washed with 10 volumes of distilled H_2_O.

For all non-covalent conjugations, 0.5 g of mNC was suspended in 1 mL of 10 mM sodium phosphate. The buffer pH was set at 5.0 and at 8.0 to study the immobilization in an acid and a basic media. For a preliminary screening, FSA (0.02 U mL^-1^) was left to immobilize for 60 min to mNC-Si, mNC-PEG, mNC-NH_2_, mNC-COOH, mNC-CHO, mNC-SH, mNC-C≡CH, mNC-N_3_, mNC-EDTA. For the formation of a covalent bond between mNC-NH_2_ and FSA, a 3-hour incubation with EDAC (1–25 mM) was carried out, followed by addition of 0.5 M NaCl to eliminate the electrostatic interactions. All immobilizations were performed at 25°C under mild orbital stirring. Final mNC-FSA conjugates were washed with buffer to remove the remaining reagents.

In this case due to the low enzymatic activity, the immobilization yield was determined from the protein concentration in the supernatant by Bradford quantification, using bovine serum albumin as a reference [44]. The retained activity was calculated from the aldol addition test analyzed by HPLC.

### Immobilization in mesoporous carriers

#### Co-IDA

Cobalt chelated agarose (Co-IDA) beads (50–150 μm, density of 10.7 g mL^-1^) were prepared from high-density metal-chelated supports (20–40 μmol divalent metal mL^-1^ gel, with IDA as the residue to chelate the metal). In order to load the metal, 100 mL of IDA-sepharose were incubated with 300 mL of 0.2 M CoCl_2_ (pH 4.7) for 12 h. After washing with distilled water to remove the excess of metal, the support was stored in EtOH 20% v v^-1^ at 4°C [45]. FSA immobilization in Co-IDA was carried out as follows: 1 mL of Co-IDA-sepharose support was suspended in 9 mL of buffer (50 mM sodium phosphate at pH 8.0 with 300 mM NaCl and 20 mM imidazole). 0.8–500 units of FSA were added to the mixture and incubated under mild agitation on a roller at 25°C for 30 minutes. The support was finally washed with buffer.

Immobilization studies were performed using an immobilized derivative of 8 U mL^-1^ support since no mass transfer limitations are detected at this enzyme load (mass transfer limitations are detected above 10 U mL^-1^).

#### MANA-agarose

FSA immobilization in MANA-agarose (50–150 μm, density of 1.07 g mL^-1^, 200 mmol amino groups mL^-1^ of carrier) was carried out as follows: 9 ml of 10 mM sodium phosphate at pH 6.0 were mixed with 1 mL of MANA-agarose gel. 0.5–380 units of FSA were added to the mixture and incubated in mild agitation on a roller at 25°C for 30 min. When no activity was detected in the supernatant, different concentrations of EDAC were added and the enzymatic mixture was incubated under mild agitation for 3 hours. Then 1 M NaCl was added and left for 1 hour. The support was finally washed with buffer.

Immobilization studies were performed using an immobilized derivative of 4 U mL^-1^ support since no mass transfer limitations are detected at this enzyme load (mass transfer limitations are detected above 6 U mL^-1^).

#### Glyoxal-agarose

The glyoxal-agarose support was prepared by etherification and oxidation of 6% cross-linked agarose beads (17 aldehyde residues per 1000 Å^2^ of gel surface), according to the protocol reported by Guisán *et al*. [46].

Immobilization of FSA was carried out as follows: 1 mL of glyoxal-agarose was mixed with 9 mL of 50 mM bicarbonate at pH 10.0. 1–380 units of FSA were added and incubated under mild agitation on a roller at 25°C for 3 hours. When no activity was detected in the supernatant, NaBH_4_ was added at a final concentration of 1 mg mL^-1^ and incubated in mild agitation for 30 minutes. Finally, the carrier was washed with buffer.

Immobilization studies were performed using an immobilized derivative of 4 U mL^-1^ support since no mass transfer limitations are detected at this enzyme load (mass transfer limitations are detected above 5 U mL^-1^ support).

#### Determination of immobilization yield and activity recovery

Immobilizations were characterized by immobilization yield (Eq 1) [47] and retained activity quantification (Eq 2). Samples of supernatant (SN) and suspension (SP) were periodically taken during the immobilization process to determine the FSA activity. In parallel, FSA activity in a blank without support was monitored over time. Once the immobilization is finished, the immobilized derivative was washed twice with the corresponding immobilization buffer and re-suspended in fresh immobilization buffer. The activity of the resuspension (RSP) was analyzed to determine the retained activity. Experiments were carried out in duplicate and the standard error was determined (S.D.).

$$\mathrm{Immobilization}\;\mathrm{yield}\;(\%)=\frac{\left(\mathrm{Initial}\;\mathrm{activity}‐\mathrm{SN}\;\mathrm{activity}\right)}{\left(\mathrm{Initial}\;\mathrm{activity}\right)}\times 100$$ (1)

$$\mathrm{Retained}\;\mathrm{activity}\;(\%)=\frac{\left(\mathrm{RSP}\;\mathrm{activity}\right)}{\left(\mathrm{Initial}\;\mathrm{activity}\right)}\times 100$$ (2)

### Synthesis of pre-D-fagomine

10 U of FSA was added to a final volume of 10 mL of reaction medium (1 U mL^-1^ reaction) containing 30 mM β-CHO, 45 mM DHA and 50 mM HEPES buffer at pH 8.0. The reaction was carried out at 25°C, under orbital stirring. Samples were taken periodically and analyzed by HPLC in order to quantify β-CHO and pre-D-fagomine concentrations.

When immobilized derivatives were used, biocatalysts were previously prepared in mNC-NH_2_, in MANA-agarose, in glyoxal- and in Co-IDA beads at 10.0 ± 1 U mL^-1^ support. These derivatives were added in the reaction mixture to reach 1 U mL^-1^ of reaction to a final reaction volume of 10 mL, except for the evaluation of the mNC-NH_2_ derivative which was performed in a final reaction volume of 1 mL. Experiments were carried out in duplicate and the standard error was determined (S.D.).

To evaluate the performance of the immobilized FSA on multiple synthesis cycles, immobilized derivatives were added to the reaction medium with a final volume of 10 mL to reach 1 U mL^-1^ reaction and after each cycle the support was recovered and washed with 50 mM HEPES buffer at pH 8.0, then new reaction medium was added. Substrate conversion and product yield were quantified.

### Cbz-aldehyde and pre-D-fagomine quantification

The concentrations of β-CHO and pre-D-fagomine were measured by HPLC analysis in a Dionex UltiMate 3000 with a variable wavelength detector. The reversed-phase column CORTECS C18+ 2.7 μm 4.6×150 mm from Waters was employed. Reaction samples were dissolved in acetonitrile. The analyses were performed by injecting 15 μL of the sample at a flow rate of 0.7 mL min^-1^, 30°C. The solvent system consisted of solvent A –0.1% (v v^-1^) trifluoroacetic acid (TFA) in H_2_O– and solvent B –0.095% (v v^-1^) TFA in acetonitrile/H_2_O 4:1 (v v^-1^)–. Samples were eluted using a gradient from 5 to 28.5% B in 0.5 min, and an isocratic elution at 28.5% B over 15 min (λ = 254 nm) [39]. Prior calibration with standards of known concentration was used for quantitative analysis of β-CHO. The standard deviation was calculated from duplicated measurements of a single sample.

The pre-D-fagomine product was characterized and confirmed by HPLC-MS analysis (S2 Fig).

## Results and discussion

### Immobilization to functionalized mNC

**Fig 2:**
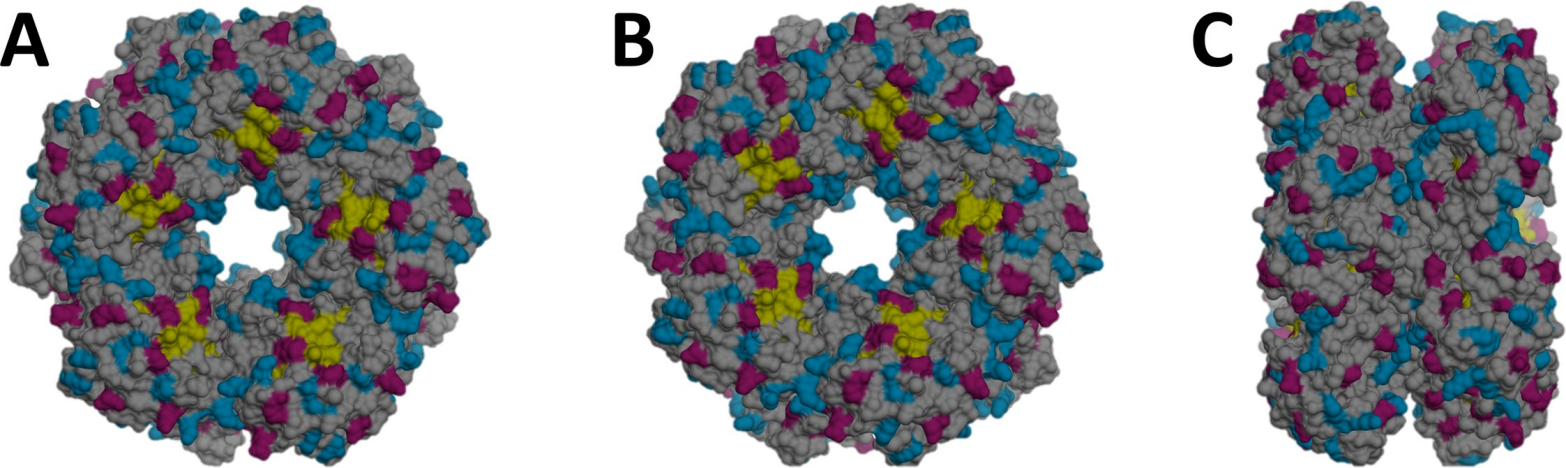
Tridimensional structure of fructose-6-phosphate aldolase (FSA). The entrances to the two active sites are located between the Lys85 from five subunits, in yellow. Acid residues are colored in red; basic residues are in blue. His-tag is not shown; not included in the file used from the Protein Data Bank (PDB) [7]. View: (A) front, (B) back, (C) side. Represented using UCSF Chimera software.

A primary screening on the immobilization of FSA onto mNC was performed by the incubation of the enzyme with functionalized mNC (-Si, -PEG, -NH_2_, -COOH, -CHO, -N_3_, -C≡CH, -EDTA). The protein concentration in the supernatant was evaluated by Bradford quantification due to an enzymatic activity too low to be quantified in an activity assay (S1 Table). The immobilization strategy was successful through both amino and carboxylic groups from the enzyme. This was caused by the enzyme structure (Fig 2): NH_2_ and COOH groups are similarly distributed and available along the FSA surface. The attachment through the amino groups of the enzyme produced a total or partial loss of the activity. This could be explained by a possible linkage between mNC-COOH and Lys85 of FSA, which has a key role in the catalysis of this enzyme [8].

In all cases (S1 Table), almost all the enzyme was released from the particles after washing with buffer (final immobilization yield <10%). Thus, their use for catalysis is not recommended without further modification steps. To that end, the well-explored EDAC chemistry approach was used to covalently bind the amine-functionalized nanoparticle clusters with the carboxyl group of the enzyme (S2 Table). EDAC at about 5 mM (2.5–10 mM range) was the pseudo-optimal concentration, leading to a final retained activity of 29%. The activity loss in comparison to the electrostatically immobilized derivative can be related to higher bond rigidity, and also to cross-linking of the enzyme. Further modifications on the active derivatives on S1 Table (for instance, reduction of the Schiff base) could enable the preparation of catalytically-valuable derivatives.

### Immobilization in mesoporous carriers

FSA immobilization was also studied by different methods using agarose-based supports. Supports based on agarose matrixes have been extensively reported as efficient carriers for enzyme immobilization [48–53]. Two different attachment strategies were tested: i) affinity interaction by means of the His-tag of the FSA and ii) covalent binding by different functional groups of the enzyme (-COOH or -NH_2_).

Co-IDA was used for studying the immobilization by affinity interaction (Table 1). Results showed high immobilization yield (>99 ± 2.1%) and retained activity (93.8 ± 3.2%). Due to the promising results, the maximum loading capacity of the support was tested obtaining an immobilized derivative with 466.7 U mL^-1^.

**Table 1:** Immobilization yield and retained activity of FSA immobilized by different methods on mesoporous supports. The immobilizations were performed at room temperature under mild agitation.

Carrier	Conditions	Method	Immobilization yield ± S.D. (%)	Retained Activity ± S.D. (%)
Co-IDA*	0.3 M NaCl, 20 mM imidazol	Affinity	>99 ± 2.1	93.8 ± 3.2
MANA-agarose**	5 mM EDAC	Covalent	80.9 ± 3.1	52.7± 2.9
	15 mM EDAC	Covalent	90.2 ± 2.1	87.8 ± 4.2
	25 mM EDAC	Covalent	93.4 ± 1.6	89.7 ± 6.1
Glyoxal-agarose***	1 mg mL^-1^ NaBH_4_	Covalent	>99 ± 0.9	53.9 ± 1.1

Immobilization buffers

*50 mM Sodium phosphate pH 8,0

**10 mM Sodium phosphate pH 6.0

***50 mM bicarbonate pH 10.0.

Immobilization by means of the -COOH groups of the biocatalyst was carried out using amino-functionalized agarose (MANA-agarose). The linkage takes place in two steps. Firstly, an ionic interaction between the positively charged amino groups of the support and the negatively charged carboxyl groups of the enzyme occurs. Then, EDAC is used as the agent to promote a covalent bond formation. Finally, the non-covalently attached enzyme is desorbed by increasing the ionic strength of the solution. Three different EDAC concentrations were tested (5, 15 and 25 mM) resulting in higher immobilization yields as the EDAC concentration is increased (Table 1). The corresponding retained activities showed that 5 mM of EDAC is not enough to promote the required covalent binding of the ionically adsorbed FSA (53%). Increasing EDAC concentration up to 15 and 25 mM leads to retained activities above 85%. Considering the immobilization yields and retained activities results, 25 mM was selected as the most suitable covalent bond promoting agent concentration for FSA immobilization in MANA-agarose. Applying these conditions, a maximum loaded immobilized derivative was obtained with 336.4 U mL^-1^, a 28% lower than the derivative obtained with Co-IDA.

When FSA was immobilized by its -NH_2_ groups, aldehyde functionalized agarose was used as a carrier (glyoxal-agarose). The linkage between the enzyme and the carrier takes place in a first step by a Schiff base formation. Then, a reducing agent (NaBH_4_) is added to form the covalent linkage by the amide bond formation. Following this methodology, all the enzyme offered to the support was immobilized (Table 1). However, the retained activity only reached 54%, indicating an activity loss during the immobilization procedure. When the maximum loading capacity of this support was studied, an immobilized derivative of 174.1 U mL^-1^ was obtained, 2.7-fold and 1.9-fold less active than the Co-IDA and the MANA-agarose derivative, respectively. Again, the low retained activity obtained by -NH_2_ linkage of the FSA to the glyoxal-agarose could be related to a covalent bond formation between a lysine located in the active center of the enzyme and the support. This non-desired interaction could lead to a decrease in the FSA activity once it is immobilized.

Other authors have reported high retained activities of FSA by using Mg_2_Al–NO_3_ (90%), Mg_2_Al (92%), Zn_2_Al (72%) or MgZnAl (88%) layered double hydroxide (LDH) as support. These results, especially for Mg_2_Al–NO_3_, Mg_2_Al, and MgZnAl, are similar to those reported in the present work for Co-IDA (93.8%) and MANA-agarose (89.7%) [35,37]. It has also been reported a successful immobilization of FSA by encapsulation of FSA-LDH in a carrageenan polymer obtaining an activity recovery of over 40% [36].

### Pre-D-fagomine synthesis by aldol addition

**Fig 3:**
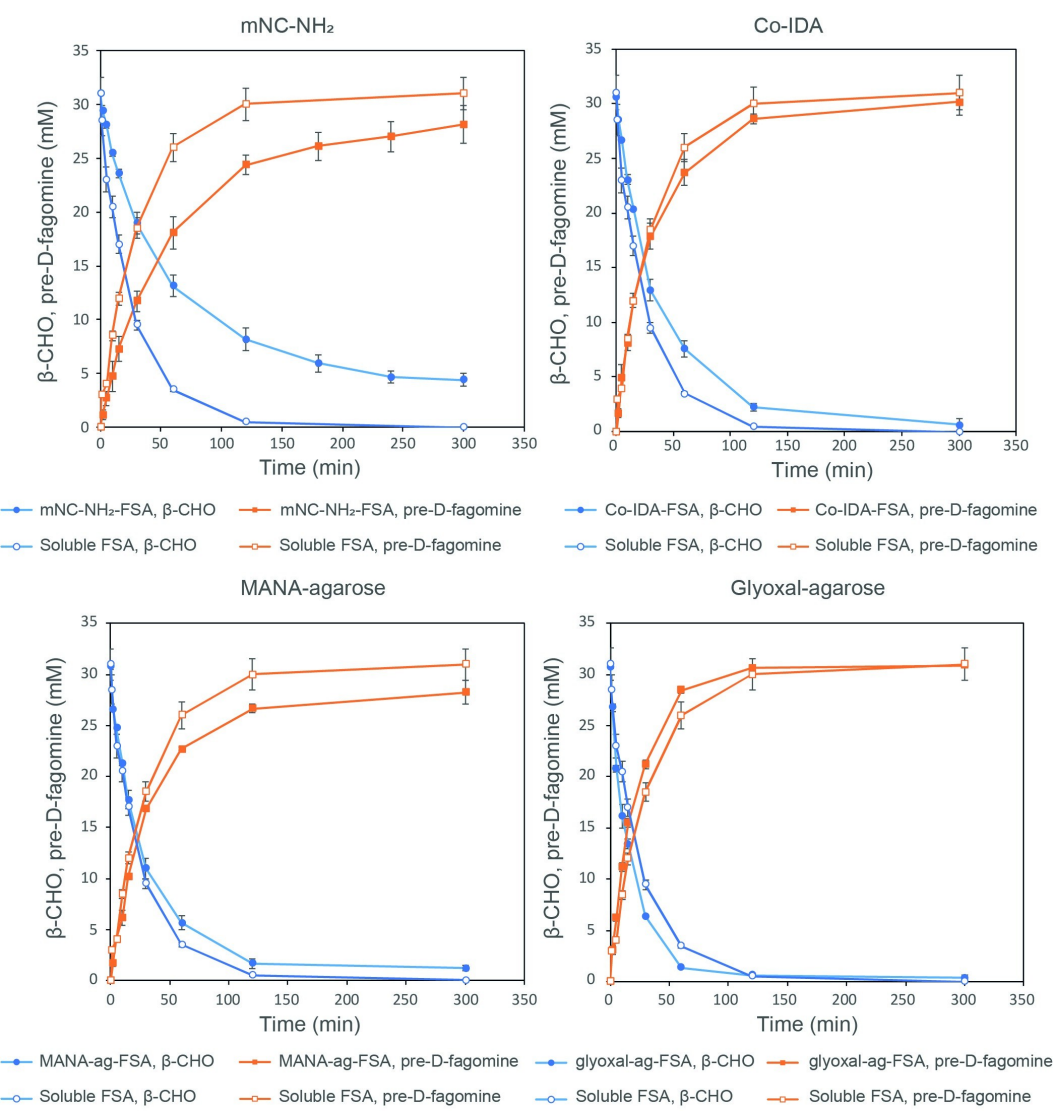
Aldol addition reaction of DHA and β-CHO (●) producing pre-D-fagomine (■) catalyzed by soluble FSA and FSA immobilized in mNC-NH_2_ (A), in Co-IDA (B), in MANA-agarose (C) and in glyoxal-agarose (D). The reaction catalyzed by soluble enzyme is represented with the dashed line in all the reaction plots. The reaction medium contained: 45 mM DHA, 30 mM β-CHO and 1 U mL^-1^ of reaction. The aldol addition was performed at 25°C and pH 8.0 and 10 mL total reaction volume (1 mL reaction volume for the mNC-NH_2_ derivative).

The synthesis of pre-D-fagomine was studied using the immobilized FSA derivatives: mNC-NH_2_, Co-IDA, MANA-agarose (25 Mm EDAC) and glyoxal-agarose. The results were compared with the performance of the reaction using soluble FSA (Fig 3, Table 2). Aiming to compare all immobilized derivatives, 1 U mL^-1^ of reaction was applied since it was the maximum load that can be achieved using the immobilized derivative with the lowest maximum retained activity, (mNC-NH_2_).

**Table 2:** Reaction yield, conversion and initial reaction rate of pre-D-fagomine synthesis catalyzed by FSA. Reactions conditions: 1 U mL^-1^, 10 mL reaction volume*, 30 mM β-CHO, 45 mM DHA and 50 mM HEPES buffer, pH 8.0, 25°C, orbital stirring.

Biocatalyst	Yield ± S.D. (%)	Conversion ± S.D. (%)	Initial reaction rate ± S.D. (mM product min^-1^)
**Soluble**	>99	>99	0.8±0.03
**mNC-NH_2_**	92±1.6	87±5.6	0.43±0.01
**Co-IDA**	96±3.6	97±1.9	0.79±0.04
**MANA-agarose**	68±2.9	95±0.8	0.64±0.04
**Glyoxal-agarose**	>99	98±0.8	1.01±0.05

*1 mL reaction volume for the mNC-NH_2_ derivative.

Soluble FSA led to full substrate conversion and > 99% yield reaching an initial reaction rate of 0.80 mM min^-1^.

Co-IDA and glyoxal-agarose derivatives showed no mass transfer limitation according to the initial reaction rates, 0.79 and 1.01 mM min^-1^, respectively. In both cases, conversion and yield were > 95%, thus performing as the soluble enzyme. Regarding MANA-agarose, even though the initial reaction rate was only 20% lower compared to the soluble enzyme and 95% conversion was achieved, a lower yield was obtained (68%). These results could be related to a side-reaction of the β-CHO and the–NH_2_ of the support leading to a Schiff base formation [39]. This side-reaction effect could be seen comparing the initial substrate reaction rates between the synthesis catalyzed by soluble and immobilized FSA (Fig 3C). The substrate concentration decreases at higher rates when MANA-agarose is used, achieving a 30% conversion after 2 minutes which corresponds to the imbalance between conversion and yield at the end of the reaction.

Regarding mNC-NH_2_ derivative, lower reaction rate and conversion were obtained (0.43 mM min^-1^ and 87%). As already mentioned, low retained activities were achieved in FSA immobilization to mNC-NH_2_ (29%), leading to an immobilized derivative with low specific activity. Therefore, high derivative load was required in the reaction mixture to reach 1 U mL^-1^, causing an increase of the media viscosity. According to other authors, the catalytic properties of enzymes immobilized in nanoparticles depend on the particle size and the media rheology since these parameters are linked to diffusion and particle interaction [29]. Therefore, the low catalytic performance of the mNC-NH_2_ derivative could be related to an increase in media viscosity.

### Reusability of FSA immobilized derivatives to perform pre-D-fagomine synthesis

**Fig 4:**
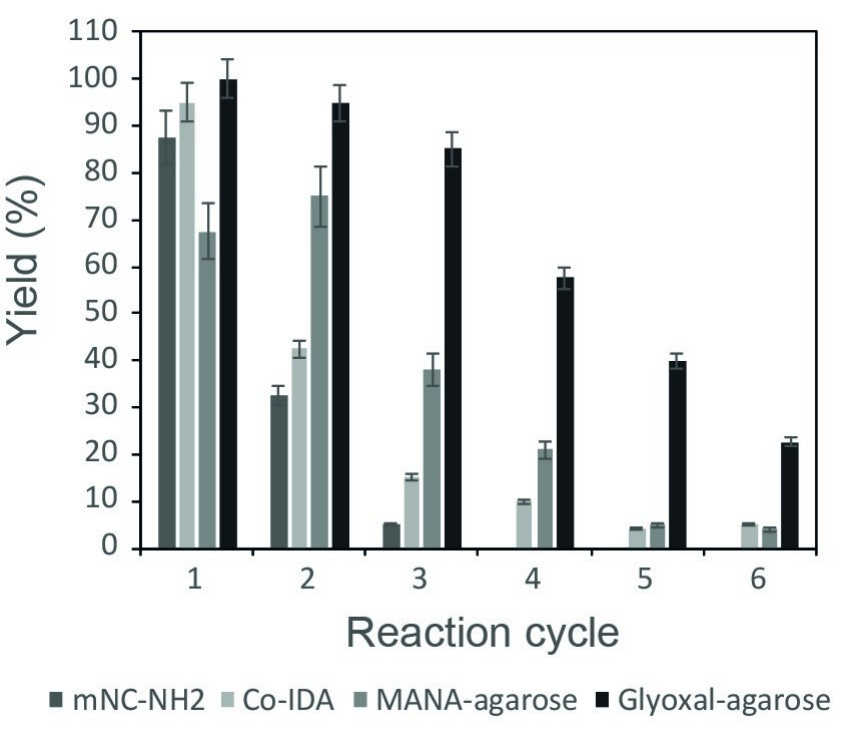
Pre-D-fagomine reaction yields catalyzed by FSA immobilized in mNC-NH_2_, Co-IDA, MANA-agarose and Glyoxal-agarose in subsequent reaction cycles. The reaction medium contained: 45 mM DHA, 30 mM β-CHO and 1 U mL^-1^ of reaction. The aldol addition was performed at 25°C and pH 8.0 for 5 hours.

One of the main advantages of enzyme immobilization is the possibility to re-use the biocatalyst in subsequent reaction cycles. Thus, the operational stability of the obtained FSA derivatives in the synthesis of pre-D-fagomine was evaluated. The obtained results are depicted in Fig 4.

FSA immobilized onto mNC-NH_2_, in addition to lead the lowest reaction rate and conversion in the first reaction cycle (Table 2), it also showed the lowest operational stability (Fig 4). The obtained yield in the second and third cycle only reached 20% and 2%, respectively and no conversion was detected in the subsequent cycles. Therefore, this derivative was discarded for further analysis.

Regarding FSA Co-IDA derivative (Fig 4), even though it showed promising results in the first cycle in terms of initial reaction rate, yield and conversion (Table 2), the operational stability was low. The reaction yield strongly decreased in the subsequent cycles reaching only 40% and 20% in the second and third cycle and lower than 10% from the fourth to the sixth reaction cycle.

When MANA-agarose was evaluated, higher operational stability than Co-IDA and mNC-NH_2_ was observed, reaching yields higher than 60% in the two first cycles with a slight decrease in the subsequent reactions (Fig 4). However, as already mentioned, the substrate reacts with the support, reason why the yield in the first cycle is lower compared to the other derivatives.

Finally, glyoxal-agarose FSA derivative stood up as the best candidate for the synthesis of pre-fagomine (Fig 4). In addition to high reaction rate, conversion and yield obtained in the first cycle, the operational stability was the highest compared to the other immobilized biocatalysts. Yields were higher than 80% in the second and third cycle and higher than 20% up to the sixth cycle.

Aiming to validate that glyoxal-agarose derivative was the best option in terms of process metrics for pre-D-Fagomine synthesis, the total product quantity (μmol of pre-D-fagomine), the total reaction yield (%) and the biocatalyst yield (μmol of pre-D-fagomine obtained by 1 U of FSA) were evaluated and compared with the performance of the soluble FSA, Co-IDA and MANA-agarose derivatives (Table 3).

**Table 3:** Total product quantity, total reaction yield and biocatalyst yield for the synthesis of pre-D-fagomine using immobilized FSA in 6 reaction cycles and soluble FSA in a single cycle. Reactions conditions: 1 U mL^-1^, 10 mL reaction volume, 30 mM β-CHO, 45 mM DHA and 50 mM HEPES buffer, pH 8.0, 25°C, orbital stirring.

Biocatalyst	Total product quantity (μmol)	Total reaction yield (%)	Biocatalyst yield (μmol product U^-1^)
**Soluble**	300.0±15	> 99	30.0±1.5
**Co-IDA**	498.6±45	27.7±9	49.9±4.6
**MANA-agarose**	667.8±37	37.1±5	66.8±3.7
**Glyoxal-agarose**	1206±43	67.0±3	120.6±4.3

According to the obtained process metrics, the use of immobilized derivatives led to a reduction of total reaction yield. However, an increase in both total product quantity and biocatalyst yield is obtained with all immobilized derivatives. Both Co-IDA and MANA-agarose led to a 1.6-fold and 2.2-fold increase in biocatalyst yield, respectively. Glyoxal-agarose resulted in the highest product amount and biocatalyst yield reaching a 4-fold increase in both parameters. Therefore, glyoxal-agarose was considered as the best immobilized derivative to perform the synthesis of pre-D-fagomine.

Other authors have also reported a decrease in reaction conversion when FSA has been immobilized and reused in other aldol condensations. FSA immobilized in Mg_2_Al–NO_3_ layered double hydroxide (LDH), reached a 70% conversion in the first cycle of the condensation of hydroxyacetone and formaldehyde and it decreased to 61% in the fourth cycle. After four reaction cycles, the FSA activity was maintained at over 87% [35]. FSA immobilized by encapsulation of FSA-LDH in a carrageenan polymer was also reported to catalyzed the aforementioned condensation maintaining over 80% of the initial activity after four cycles reaching a 60% conversion in the first reaction and 50% in the next reaction cycles [36].

## Conclusions

The mutant FSA A129S efficiently catalyzes the synthesis of pre-D-fagomine by the aldol addition of DHA and β-CHO. An immobilization screening of FSA on mNC and agarose-based carriers was performed aiming to select the most suitable biocatalyst to be reused in consecutive aldol addition reactions. The results showed that FSA immobilized on Co-IDA beads was the best biocatalyst for the synthesis of pre-D-fagomine in one unique reaction. However, when biocatalysts were recovered and reused in several reaction cycles, the biocatalyst immobilized in glyoxal-agarose was the best candidate, especially due to its high operational stability. The glyoxal-agarose derivative could be reused in 6 consecutive reaction cycles reaching a 4-fold biocatalyst yield improvement compared to the soluble enzyme.

## Supporting information

S1 Fig SDS-PAGE gel from FSA sample purified using Co-IDA agarose. Lane 1: Precision Plus Protein^TM^ standard (10–250 kDa, Bio-Rad Laboratories). Lane 2: FSA band of approximately 23 kDa. Image Lab© (Bio-Rad) was used for band determination.
(PDF)

S2 Fig MS–ESI+ (Na^+^) spectra of preFagomine (m/z = 320.1106). (PDF)

S3 Fig The relation between percentage of FSA activity and product formation in the activity test of the DHA aldol addition to β-CHO. %FSA stands for the initial active amount of FSA used in the screening of FSA onto mNC (0.02 U ml^-1^).
(PDF)

S1 Table Screening of the FSA immobilization onto functionalized mNC. IY: immobilization yield, RA: retained activity. No NaCl was added in any case; n.d.: not determined; S.D. is calculated from more than two replicas in each case.
(PDF)

S2 Table Screening of the EDC concentration for the covalent immobilization of FSA onto mNC-NH2. Immobilization conditions: 3 h in 10 mM phosphate buffer (pH 5.0), 25°C. Washing corresponds to two 5-minute consecutive washing cycles with 0.25 M NaCl.
(PDF)

S1 Raw images (TIF)
