# Supplementary material for: Synthesis of a precursor of D-fagomine by immobilized fructose-6-phosphate aldolase
Source: PLoS One. 2021 Apr 22;16(4):e0250513. doi: 10.1371/journal.pone.0250513 (PMC8062046; doi:10.1371/journal.pone.0250513)
Supplement: S1 Fig — Lane 1: Precision Plus ProteinTM standard (10–250 kDa, Bio-Rad Laboratories). Lane 2: FSA band of approximately 23 kDa. Image Lab© (Bio-Rad) was used for band determination. (PDF) [file pone.0250513.s001.pdf]

**FSA purification**

Recombinant FSA A129S was produced and purified in *Escherichia coli* BL21 (DE3) from plasmid pET22-fsaA, which was generously supplied by Dr. Clapés from the Biotransformation and Bioactive Molecules Research Group (IQAC-CSIC, Barcelona) [37]. The cell pellets were recovered from the reactor by centrifuging the culture medium at 1000 rpm for 45 minutes, then the biomass was disrupted in a mechanical device (Constant Cell Disruption Systems, Northamptonshire, UK) using a pressure of 2.76 kBar. Recombinant FSA was purified by a two-step process using an AKTA pure 150 (GE Healthcare Lifescience, Chicago, USA). The first stage consisted of an affinity purification taking advantage of the fact that the enzyme is produced fused to a tail of histidines, and the second stage was a desalting process to eliminate the high concentration of salts from the buffer in which the pure enzyme was recovered. The measured specific activity of FSA after purification was 15.0 U $mg^{-1}$ FSA.

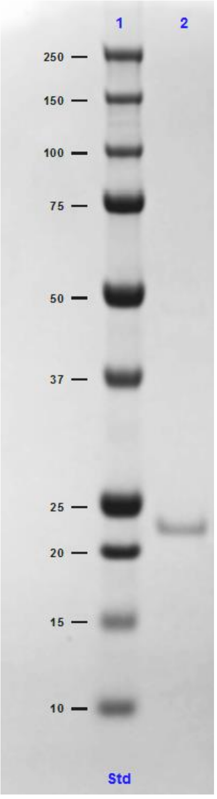


**S1 Fig**. **SDS-PAGE gel from FSA sample purified using CoIDA agarose**. Lane 1: Precision Plus Protein™ standard (10-250 kDa, Bio-Rad Laboratories). Lane 2: FSA band of approximately 23 kDa. Image Lab© (Bio-Rad) was used for band determination.
