# Supplementary material for: Synthesis of a precursor of D-fagomine by immobilized fructose-6-phosphate aldolase
Source: PLoS One. 2021 Apr 22;16(4):e0250513. doi: 10.1371/journal.pone.0250513 (PMC8062046; doi:10.1371/journal.pone.0250513)
Supplement: S2 Fig — (PDF) [file pone.0250513.s002.pdf]

Analysis of preFagomine

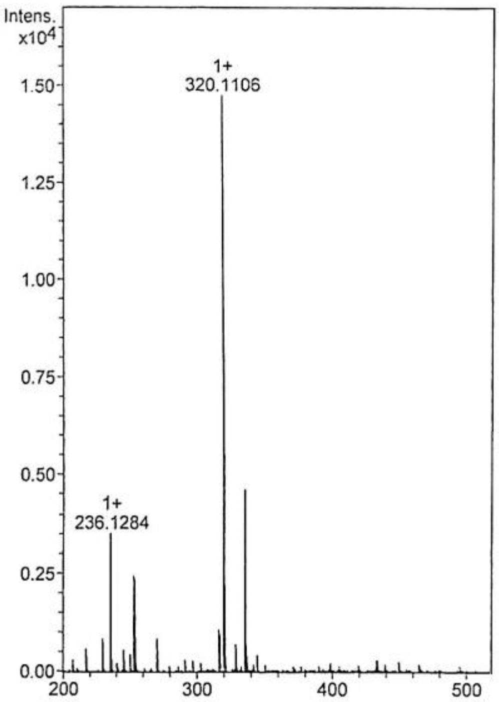

S2 Fig. **MS–ESI+ (Na^+) spectra of preFagomine** (m/z = 320.1106).

For the preparation of preFagomine sample, 20 mM β-CHO, 30 mM DHA, and 0.13 mg FSA mL^{-1} were incubated for 120 min in distilled water (no buffer was used to reduce the amount of salts for the analyses). Figure shows the HPLC-MS results that confirm the proposed preFagomine structure. These analyses were performed at *Servei d'Anàlisi Química, UAB.*

preFagomine: colorless; MS-ESI+ (Na^+): *m/z* = 320.1106, calculated for $C_{14}H_{19}NO_6$: 320.1105.
