# Supplementary material for: Synthesis of a precursor of D-fagomine by immobilized fructose-6-phosphate aldolase
Source: PLoS One. 2021 Apr 22;16(4):e0250513. doi: 10.1371/journal.pone.0250513 (PMC8062046; doi:10.1371/journal.pone.0250513)
Supplement: S3 Fig — %FSA stands for the initial active amount of FSA used in the screening of FSA onto mNC (0.02 U ml-1). (PDF) [file pone.0250513.s003.pdf]

Activity test (HPLC)

This test consists in the target aldol addition of DHA to β-CHO catalyzed by FSA, measuring the formation of preFagomine on HPLC. This test shows great linearity at within the experimental range of FSA concentration.

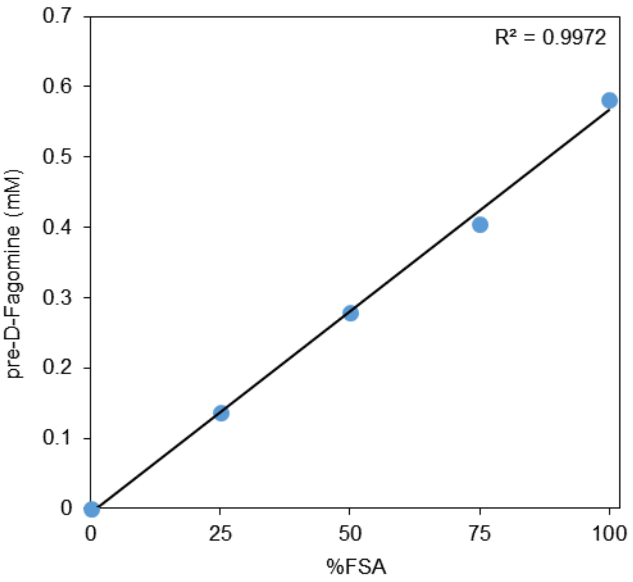

S3 Fig. The relation between percentage of FSA activity and product formation in the activity test of the DHA aldol addition to β-CHO. %FSA stands for the initial active amount of FSA used in the screening of FSA onto mNC (0.02 U ml^{-1}).
