# Supplementary material for: Synthesis of a precursor of D-fagomine by immobilized fructose-6-phosphate aldolase
Source: PLoS One. 2021 Apr 22;16(4):e0250513. doi: 10.1371/journal.pone.0250513 (PMC8062046; doi:10.1371/journal.pone.0250513)
Supplement: S1 Table — IY: immobilization yield, RA: retained activity. No NaCl was added in any case; n.d.: not determined; S.D. is calculated from more than two replicas in each case. (PDF) [file pone.0250513.s004.pdf]

Immobilization of FSA onto functionalized mNC

FSA was immobilized onto differently functionalized mNC, analyzing how the reactivity, the abundance and the location of the functional groups of the enzyme affect the binding efficiency and the final activity of the derivative.

Any type of immobilization forming a bond or interaction strong enough to retain the enzyme attached to the particle during the target reaction (aldol addition) was desired: (a) physical adsorption onto mNC-Si, (b) Schiff base formation between mNC-CHO and amino groups from FSA, (c) covalent bond between amines and carboxylic groups from the particle (mNC-NH_2 and mNC-COOH) or the enzyme, and (d) binding trough cysteines from the enzyme and mNC-SH. Other particles were also tested: mNC-PEG as a control due to the presence of PEG in all particles (≈17 mol. %), mNC-N_3 and mNC-C≡CH were initially aimed to do click chemistry (but modification of FSA was not successful), mNC-EDTA that showed binding to the enzyme through the carboxyl groups (also tested to bind the His-tag from the enzyme).

The immobilization screening was carried out by protein quantification (Bradford methodology) of the supernatants after incubation of the enzyme and the particles. The most promising nine derivatives (yield > 50 %) were tested in terms of activity (enzymatic assay: aldol addition). After the activity screening, five derivatives were selected (activity > 50 %), and the bond strength was tested: the immobilized derivatives were washed with buffer and resuspended in fresh buffer. Almost all the enzyme was released to the supernatant with a final immobilization yield of <10%. To overcome this issue, a covalent bond between mNC-NH_2 and the carboxylic groups from FSA was aimed using EDC chemistry.

Two pH conditions were tested (pH 5 and 8) aiming to modify the electric charge of the enzyme. However, a clear trend depending on pH was not observed probably due to unspecific interactions between the enzyme and the support. It should be also taken into account that electrostatic forces are not the only interactions driving these linkages.

S1 Table: Screening of the FSA immobilization onto functionalized mNC. IY: immobilization yield, RA: retained activity. No NaCl was added in any case; n.d.: not determined; S.D. is calculated from more than two replicas in each case.

Reactive group		pH 5.0		pH 8.0	
mNC	FSA	IY (%)	RA (%)	IY (%)	RA (%)
Si (OH)	OH	77.5 ± 7.6	59.5 ± 5.6	20.5 ± 14.6	
PEG	OH	15.8 ± 19.3		6.1 ± 10.0	
NH_2	COOH	>99 ± 19.5	61.0 ± 12.8	>99 ± 15.8	21.0 ± 3.9
COOH	NH_2	>99 ± 11.3	2.1 ± 2.4	9.8 ± 15.1	
CHO	NH_2	>99 ± 17.1	60.0 ± 14.3	67.2 ± 5.6	9.8 ± 2.8
N_3	n.d.	88.8 ± 11.4	94.0 ± 20.8	6.5 ± 8.3	
SH	SH	<1 ± 5.2		<1 ± 2.8	
C≡CH	OH	26.1 ± 5.9		<1 ± 6.1	
EDTA	NH_2	>99 ± 12.0	20.0 ± 9.3	86.2 ± 7.1	55.5 ± 3.1
