# Supplementary material for: Synthesis of a precursor of D-fagomine by immobilized fructose-6-phosphate aldolase
Source: PLoS One. 2021 Apr 22;16(4):e0250513. doi: 10.1371/journal.pone.0250513 (PMC8062046; doi:10.1371/journal.pone.0250513)
Supplement: S2 Table — Immobilization conditions: 3 h in 10 mM phosphate buffer (pH 5.0), 25°C. Washing corresponds to two 5-minute consecutive washing cycles with 0.25 M NaCl. (PDF) [file pone.0250513.s005.pdf]

Analysis of the retained activity of FSA onto mNC-NH_2 (covalent)

S2 Table. Screening of the EDC concentration for the covalent immobilization of FSA onto mNC-NH_2. Immobilization conditions: 3 h in 10 mM phosphate buffer (pH 5.0), 25 ºC. Washing corresponds to two 5-minute consecutive washing cycles with 0.25 M NaCl.

EDC (mM)	Retained activity ± S.D. (%)	
	Prior washing	After washing
0	58.3 ± 0.4	3.67 ± 0.7
1	58.7 ± 0.6	16.17 ± 0.4
2.5	63.4 ± 3.3	29.8 ± 0.9
10	68.1 ± 1.0	28.3 ± 1.3
25	27.8 ± 3.3	19.4 ± 1.1
