# Supplementary figures and images for: Synthesis of a precursor of D-fagomine by immobilized fructose-6-phosphate aldolase
Source: PLoS One. 2021 Apr 22;16(4):e0250513. doi: 10.1371/journal.pone.0250513 (PMC8062046; doi:10.1371/journal.pone.0250513)

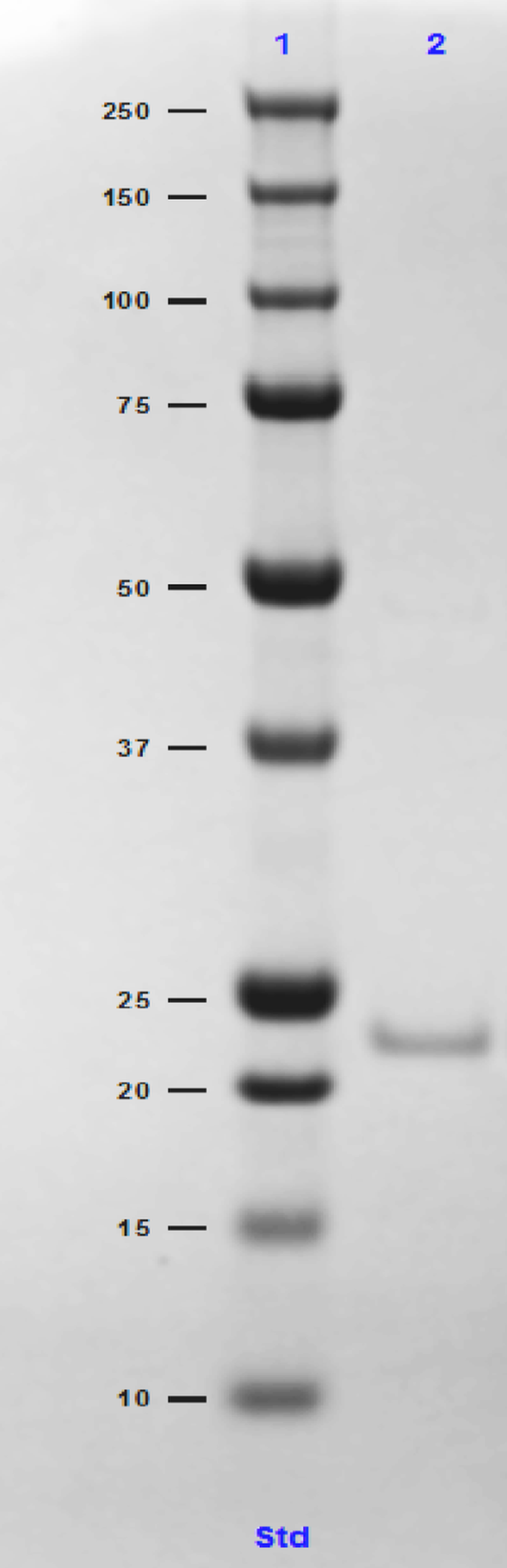

Supplement: S1 Raw images — (TIF) [file pone.0250513.s006.tif]
